# Transcription of a *cis*-acting, Noncoding, Small RNA Is Required for Pilin Antigenic Variation in *Neisseria gonorrhoeae*

**DOI:** 10.1371/journal.ppat.1003074

**Published:** 2013-01-17

**Authors:** Laty A. Cahoon, H. Steven Seifert

**Affiliations:** Department of Microbiology-Immunology, Northwestern University Feinberg School of Medicine, Chicago, Illinois, United States of America; Faculté de Médecine Paris Descartes, site Necker, France

## Abstract

The strict human pathogen *Neisseria gonorrhoeae* can utilize homologous recombination to generate antigenic variability in targets of immune surveillance. To evade the host immune response, *N. gonorrhoeae* promotes high frequency gene conversion events between many silent pilin copies and the expressed pilin locus (*pilE*), resulting in the production of variant pilin proteins. Previously, we identified a guanine quartet (G4) structure localized near *pilE* that is required for the homologous recombination reactions leading to pilin antigenic variation (Av). In this work, we demonstrate that inactivating the promoter of a small non-coding RNA (sRNA) that initiates within the G4 forming sequence blocks pilin Av. The sRNA promoter is conserved in all sequenced gonococcal strains, and mutations in the predicted transcript downstream of the G4 forming sequence do not alter pilin Av. A mutation that produces a stronger promoter or substitution of the *pilE* G4-associated sRNA promoter with a phage promoter (when the phage polymerase was expressed) produced wild-type levels of pilin Av. Altering the direction and orientation of the *pilE* G4-associated sRNA disrupted pilin Av. In addition, expression of the sRNA at a distal site on the gonococcal chromosome in the context of a promoter mutant did not support pilin Av. We conclude that the DNA containing the G-rich sequence can only form the G4 structure during transcription of this sRNA, thus providing a unique molecular step for the initiation of programmed recombination events.

## Author Summary

To evade the host immune response, pathogens have evolved mechanisms to provide genetic diversity in targets of immune surveillance. Organisms that express these diversification systems are under strong evolutionary pressure to provide subpopulations of preexisting variants and often rely on cellular recombination machinery to catalyze dedicated high-frequency reactions without disturbing genome integrity. Previously, we defined a guanine quartet (G4) structure in the strict human pathogen *Neisseria gonorrhoeae* that is required for initiating the homologous recombination reactions leading to pilin antigenic variation (Av). G4 structures have been implicated in many biological processes, however the mechanisms allowing their formation within a chromosome have not been elucidated. In this work, we show a direct link between transcription of a small RNA (sRNA) that initiates within the G4 structure forming sequence and pilin Av and conclude that the process of transcription is necessary for G4 structure formation. sRNAs have emerged as important regulatory molecules in both eukaryotes and prokaryotes, and this is a novel activity of a sRNA in a bacterium. We anticipate that the reliance of G4 structure formation on transcription is a mechanism used by other biological systems that rely on this alternative DNA structure.

## Introduction

*Neisseria gonorrhoeae* is an obligate human pathogen and the causative agent of the sexually transmitted infection gonorrhea. Gonococci generally infect the urogenital tract and the infection typically presents as urethritis in men and cervicitis in women, but many women can be asymptomatic carriers [1]. The *N. gonorrhoeae* type IV pilus is essential for establishing infection [2]. Pili assist in epithelial adherence, gonococcal cell aggregation, and also mediate twitching motility [3]–[5]. Protective immunity never develops, partially because the bacterium can evade host immune selection by varying the expression of surface antigens including lipooligosaccharides, the opacity family of outer membrane proteins, and pili [6]–[10].

Pilin antigenic variation (Av) is a high frequency diversification system that operates via a specialized recombination pathway, and utilizes enzymes that participate in general recombination and repair pathways as well as enzymes that do not participate in either pathway [11]. *N. gonorrhoeae* possesses one pilin expression locus (*pilE*) and up to 19 silent pilin loci (*pilS*) residing in up to 6 discrete locations in the genome [12]. Pilin Av occurs as a result of non-reciprocal DNA recombination between any *pilS* copy and *pilE*, leading to the expression of a new variant protein [7]. These new variants can be fully functional, poorly expressed or not expressed, and when the pilus is not expressed or poorly expressed there is an obvious change in colony morphology on solid growth medium [13]. The difference between piliated and nonpiliated colony morphologies thus allows the ability of a strain to undergo pilin (Av) to be easily assayed [14].

A transposon-based genetic screen previously identified insertions in a DNA region upstream of *pilE* that blocked pilin Av, but did not alter pilin expression [15], [16]. A subsequent targeted genetic screen in this region identified 11 GC base pairs that when individually mutated completely blocked pilin Av, and a 12^th^ GC base pair that when mutated retained residual pilin Av activity [11], [17]. Mutation of an adjacent 13^th^ GC base pair had no effect on pilin Av but mutation of this GC base pair in addition to the 12^th^ resulted in a complete loss of pilin Av, suggesting that the 13^th^ GC base pair could partially substitute for the 12^th^ [11], [17]. The organization of these 12 GC base pairs conforms to a guanine quadruplex or guanine quartet (G4) forming sequence [17]. Biophysical studies demonstrated that this G-rich sequence formed a G4 structure, and mutations that block pilin Av also inhibited structure formation [17]. Growth of *N. gonorrhoeae* on N-methyl mesoporphyrin IX, a compound that specifically binds G4 structures but not double or single stranded DNA [18], decreased the frequency of pilin Av [17]. Furthermore, point mutations in *N. gonorrhoeae* that block pilin Av and G4 structure formation prevented single stranded nicks from being detected in both the G4 forming sequence and complement strand [17]. The results from these studies suggested that for pilin Av to occur, the *pilE* G4 sequence must form a G4 structure [17]. In order for a G4 structure to form, duplex DNA must first be converted into single stranded DNA. In this study, we have now established that pilin Av absolutely requires transcription of a non-coding sRNA which originates within the *pilE* G4 sequence, thus providing a mechanism for the initiation of this conversion event.

## Results

### Analysis of the pilin expression locus (*pilE*) guanine quartet (G4)-associated small RNA (sRNA) and the effects of mutations on pilin antigenic variation (Av)

**Figure 1:**
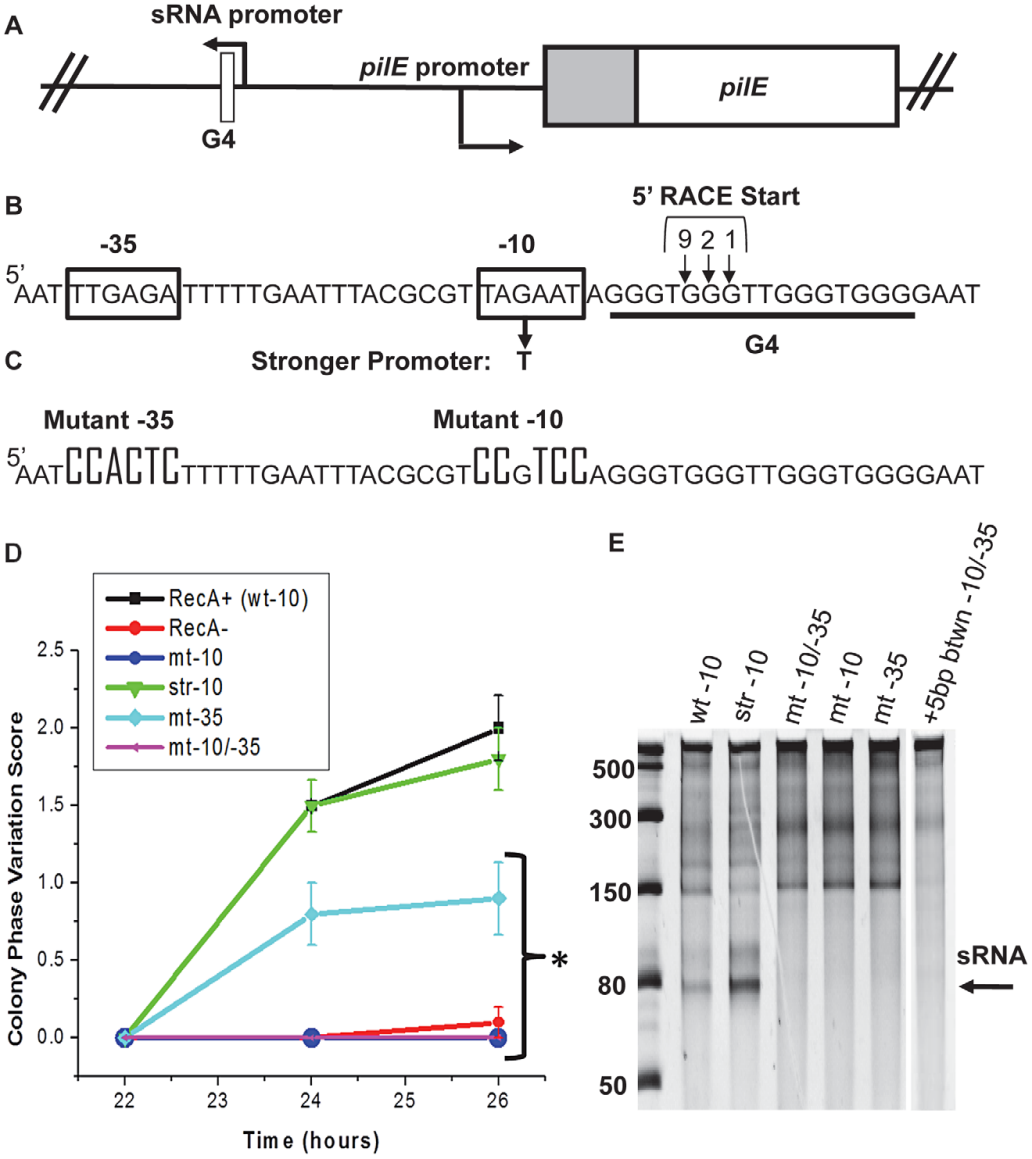
Analysis of the *pilE* G4-associated sRNA and effect on pilin Av. **A.** Chromosomal map of the pilE G4-associated sRNA locus. In gonococci, the G4-associated sRNA promoter is located upstream of *pilE* in the opposite orientation of transcription and is adjacent to the *pilE* G4. **B.** Predicted sRNA promoter sequence. Shown are the predicted sRNA −10 and −35 promoter sequences (boxed), the G to T mutation that makes a stronger promoter, and the predicted start sites detected by 5′RACE located within the *pilE* G4 forming sequence (underlined). Bases numbered and arrowed indicate the number of 5′RACE transformants showing the arrowed base as a start site. **C.** *pilE* G4 sRNA promoter mutagenesis. Knock-out mutation of the −10 and −35 promoter elements are shown **D.** Kinetic pilus-dependent colony phase variation assay. This standard assay measures the average number of visible pilus-dependent colony morphology changes occurring over time and is a surrogate measure for pilin Av [16]. Mutation of the −10 (mt-10) and/or −10 & −35 (mt-10/-35) promoter elements causes a loss in phase variation (these lines overlap), whereas mutation of the −35 promoter element alone results in an intermediate phenotype. *P*<0.05 as determined by two-tailed Student's T-test indicated by an asterisk. Bacteria having a stronger −10 promoter (str −10) element show the same levels of phase variation as the parental strain. Error bars represent the standard error of the mean for 10 colonies. A representative assay of *n* = 4 is shown. **E.** *In vitro* transcription products initated from sRNA promoter sequences. Shown is a 14% denaturing polyacrylamide gel containing *in vitro* transcribed RNA products using *Escherichia coli* RNA polymerase. Transcription was initiated from the wild-type promoter (wt-10), str-10, mt −10/−35, mt-10, mt −35, and a promoter that contains an insertion of 5 base pairs (bps) between the −10 and −35 elements (+5 bp btwn −10/−35). The RNA ladder is labeled in bases. The location of the sRNA transcript is shown by the arrow. The higher molecular weight bands represent irrelevant transcription products.

The region upstream of *pilE* that contains the *pilE* G4 is devoid of predicted genes and open reading frames. However, we identified a putative promoter sequence adjacent to the *pilE* G4 [19] (Fig. 1A & B). The most conserved bases of the −10 element [20], all the bases in the −35 element, and the relative location of this putative promoter to the *pilE* G4 are conserved in all sequenced *N. gonorrhoeae* strains and *Neisseria meningitidis* strains, except *N. meningitidis* strain FAM18, which expresses a different pilin class that cannot antigenically vary and does not have a *pilE* G4 sequence [21], [22].

To test whether the putative *pilE* G4-associated promoter was functional in *N. gonorrhoeae*, 5′ Rapid Amplification of cDNA Ends (5′RACE) adapted for bacterial small RNAs, was used to identify transcripts associated with the *pilE* G4 [23]. 5′RACE analysis detected a transcript initiating within the *pilE* G4 forming sequence and three adjacent start sites were identified (Fig. 1B). The existence of this RNA was confirmed by RNAseq analysis of *N. meningitidis* strain alpha14 (N. Heidrich and J. Vogel, personal communication). Moreover, this RNAseq analysis showed that levels of the *pilE* G4-associated sRNA in *N. meningitidis* are 125× lower than the corresponding *pilE* mRNA transcript suggesting that this sRNA is non-abundant (N. Heidrich and J. Vogel, personal communication). We have been unable to reliably detect this sRNA molecule by Northern Blot using conditions that allow other sRNA molecules to be detected (data not shown).

To determine whether the *pilE* G4-associated sRNA is required for pilin Av, promoter loss-of-function mutants were constructed and analyzed by the kinetic pilus-dependent colony morphology phase variation assay, which is a surrogate measure for pilin Av [16] (Fig. 1C & D). Mutation of the −10 sequence or both the −10 and −35 promoter elements caused a complete block of pilin Av, suggesting that transcription of the *pilE* G4-associated sRNA is required for pilin Av (Fig. 1D). Point mutations in the −10 element, a 5 bp insertion between the −10 and −35 elements, or mutation of the −35 element showed decreased pilin Av, suggesting that the level of transcription correlates with the amount of pilin Av (Fig. 1D and Fig. S1A & B). We also tested whether pilin Av was enhanced by substitution of the −10 element with a stronger promoter that was closer to the consensus sequence, but there was no change in the levels of pilin Av [20] (Fig. 1B & D). 5′-RACE analysis of the −10/−35 promoter mutant resulted in a loss of the 5′RACE product (data not shown). Taken together, these results are consistent with a model where pilin Av requires transcription of a sRNA which originates in the *pilE* G4 sequence, but that transcriptional initiation is not a rate limiting step in this process. However, we cannot ascertain how much more the stronger promoter contributes to transcription in *N. gonorrhoeae*.

**Figure 2:**
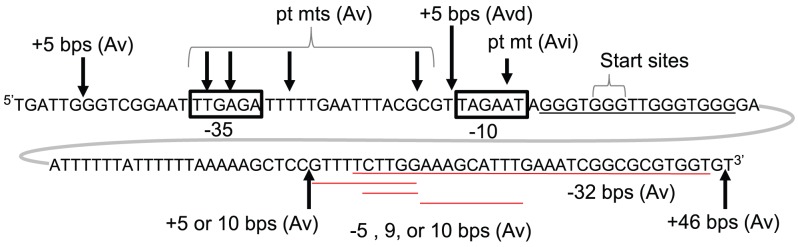
Genetic analysis of *pilE* G4-associated sRNA. The *pilE* G4-associated sRNA promoter (boxed), start site (+1) and surrounding sequence are shown. Arrows indicate the location of insertions (+) and point mutations (pt mts) while deletions (−) are underlined in red. Whether these mutations resulted in wild-type levels of pilin Av (Av), an Av deficient (Avd), or an Av intermediate (Avi) phenotype is also indicated. These Av phenotypes were determined by a pilus-dependent colony phase variation kinetic assay [16] which is shown in Fig. S1.

Not surprisingly, a 5 bp insertion upstream of the −35 element, which is not part of the sRNA promoter, had no effect on pilin Av. However 5, 10, and 46 bp insertions downstream of the G4 forming sequence, but within the predicted sRNA transcript, also had no effect on pilin Av (Fig. 2, and Fig. S1B, C & E). Furthermore, deletion of 5, 9, 10 and 32 bps [15], which are also potentially part of the sRNA transcript, had no effect on pilin Av (Fig. 2 and Fig. S1C, D & F). Since these insertions and deletions downstream of the *pilE* G4 have no effect on pilin Av, and there is no ribosome binding site or open reading frame within the sRNA sequence, we conclude that the active form of this sRNA is non-coding.

### The *pilE* G4-associated sRNA promoter and it's *in vitro* transcription profile

We further characterized the sRNA promoter using *in vitro* transcription with *Escherichia coli* sigma70 RNA polymerase (Fig. 1E). A 74 bp run-off transcript was produced from a 180 bp double stranded DNA template having the wild-type and stronger promoters, where the stronger promoter showed increased transcript levels (Fig. 1E). These results are consistent with a lack of a transcriptional stop sequence within the fragment. No transcript was observed when the −10 element and/or the −35 element were mutated or when 5 bps of heterologous sequence was inserted between the −10 and −35 sequences (Fig. 1E). Primer extension analysis of cDNA synthesized from *in vitro* transcribed RNA confirmed the products initiated from the wild-type and stronger promoters, but did not detect a product initiated from the mutated promoter (Fig. S2). These transcription profiles are consistent with the pilin Av defects observed in gonococci containing these mutations.

### Transcription of the *pilE* G4-associated sRNA acts upstream of recombination factors involved in pilin Av

RecA, RecG, and RuvB are recombination factors required for pilin Av [16], [24], [25]. RecA is a DNA recombinase, RecG is a 3′ to 5′ helicase involved in branch migration of Holliday junctions and RuvB along with RuvA is a 5′ to 3′ helicase that is also involved in branch migration of Holliday junctions. Previously, the Holliday junction processing double mutant *recG/ruvB* was found to be synthetically lethal upon *recA* induction in gonococci [25]. This lethal phenotype was rescued by a *pilE* G4 mutation that does not allow formation of the G4 structure which demonstrated that formation of the G4 structure was upstream of RecG, RuvB, and RecA during pilin Av [17].

To determine whether transcription of the *pilE* G4-associated sRNA occurs before the action of RecG, RuvB, and RecA, a −10/−35 G4-associated sRNA promoter mutation was introduced into the *recG/ruvB* double mutant, *recA* inducible strain. Gonococcal colonies in the absence of RecA induction do not show a growth defect because the bacteria cannot undergo pilin Av (Fig. S3A). Upon RecA induction, the −10/−35 promoter mutation rescues the synthetically lethal phenotype of the *recG/ruvB* double mutant showing that transcription of the *pilE* G4-associated sRNA, like formation of the G4 structure, acts before RecG, RuvB and RecA during pilin Av (Fig. S3B).

### A heterologous promoter can substitute for the *pilE* G4-associated sRNA promoter

**Figure 3:**
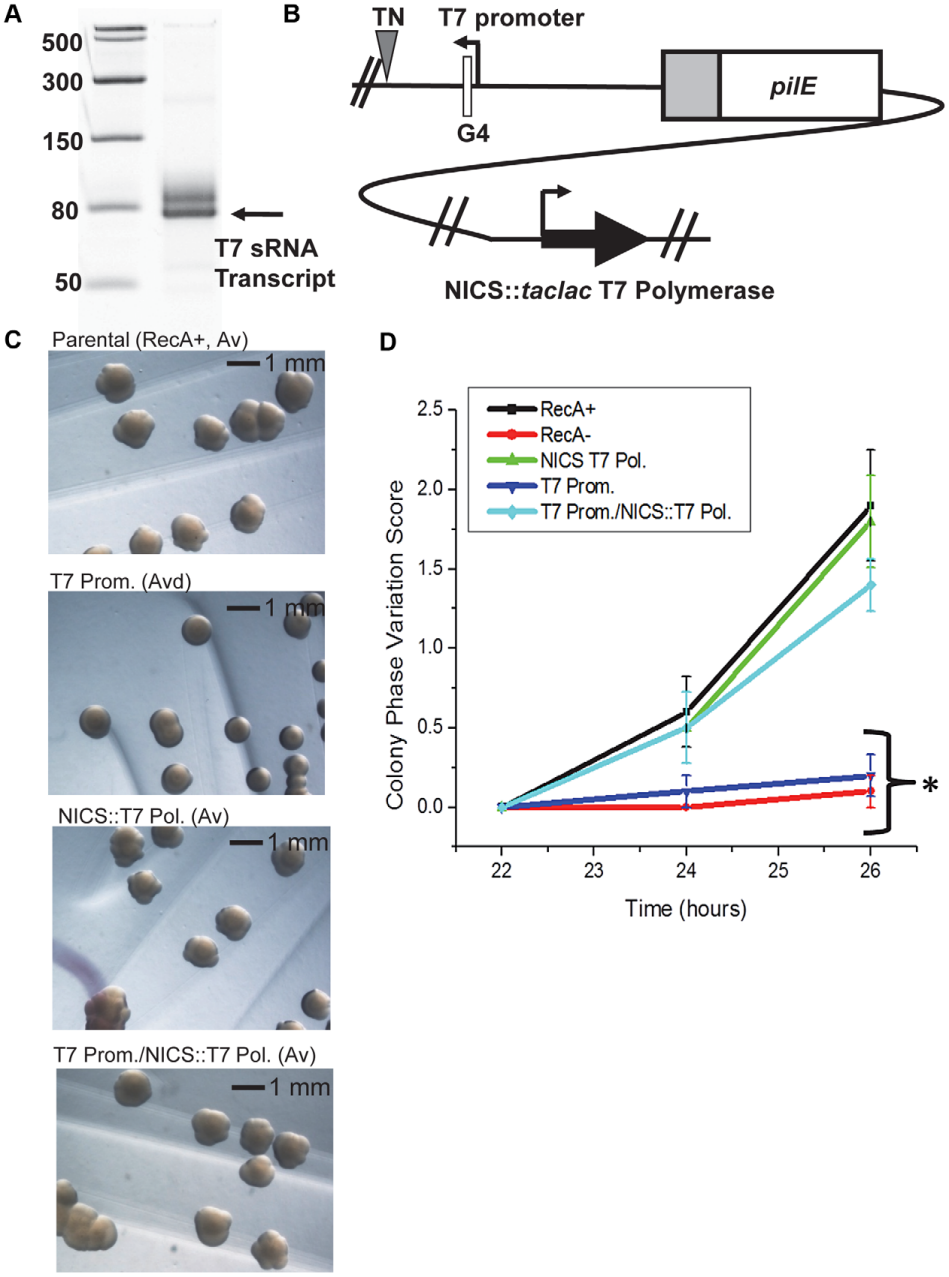
A phage promoter can substitute for the *pilE* G4-associated sRNA promoter. **A.** *In vitro* transcription. Shown is a 14% denaturing polyacrylamide gel containing T7 RNA polymerase-transcribed RNA products from a template containing the minimal T7 promoter element. The RNA ladder is labeled in bases. The higher molecular weight band is most likely an alternative start site directed by the T7 polymerase. **B.** The T7 promoter/T7 RNA polymerase strain. T7 RNA polymerase was cloned downstream of a dual *taclac* promoter which allows induction with IPTG [26]. The construct was transformed into a strain where the *pilE* G4 sRNA −10 promoter element was replaced with a minimal T7 promoter and inserted into the neisserial intergenic complementation site (NICS). [A triangle indicates a transposon (TN) encoding kanamycin resistance and was used as a marker for transformation to create the sRNA T7 promoter mutant]. **C.** Colony morphology of the T7 promoter and/or T7 polymerase strains. Colonies were grown for 46 hours with IPTG. The parental strain shows an Av phenotype, a strain expressing only T7 at the NICS (NICS T7 Pol.) has an Av phenotype, a strain where the G4 sRNA −10 element is replaced by the minimal T7 promoter (T7 Prom.) shows an Av-deficient (Avd) phenotype that is rescued with the expression of T7 polymerase (T7 Prom./NICS T7 Pol.). **D.** Kinetic pilus-dependent colony phase variation assay. Expression of T7 polymerase at the NICS (NICS T7 Pol.) does not affect phase variation whereas replacement of the G4 sRNA promoter with a minimal T7 promoter (T7 Prom.) causes a decrease in phase variation which is rescued by expression of T7 polymerase at the NICS (T7 Prom./NICS T7 Pol.). *P*<0.05 as determined by two-tailed Student's T-test (asterisks). Error bars represent the standard error of the mean for 10 colonies. A representative assay of *n* = 4 is shown.

To confirm that the pilin Av defects observed in the *pilE* G4-associated sRNA promoter mutants were a direct consequence of transcriptional defects at the *pilE* G4 locus and to test whether RNA polymerase was playing an active role in pilin Av, we expressed the *pilE* G4-associated sRNA with a T7 promoter and T7 RNA polymerase. First, the sRNA −10 promoter element was replaced with a minimal T7 promoter and tested for the ability to drive transcription using T7 RNA polymerase *in vitro* (Fig. 3A). Next, T7 RNA polymerase was inserted downstream of a *lac* promoter at the neisserial intergenic complementation site (NICS) [26] in a gonococcal strain where the *pilE* G4-associated sRNA −10 promoter element was replaced with a minimal T7 promoter (Fig. 3B). Colony morphologies examined after 46 hours of growth demonstrated that expression of T7 RNA polymerase alone in the parental strain (T7 Pol.) does not affect pilin Av, whereas replacement of the sRNA −10 promoter element with a minimal T7 promoter (T7 Prom.) produced an Av deficient (Avd) phenotype because colonies showed a stable piliated morphology after many generations (Fig. 3C). This Avd phenotype was restored to wild-type levels of pilin Av by induction of T7 RNA polymerase expression with IPTG (T7 Prom./NICS::T7 Pol.) (Fig. 3C). These observations were confirmed by the pilus-dependent kinetic colony phase variation assay (Fig. 3D). Since only T7 RNA polymerase can restore pilin Av in the sRNA minimal T7 promoter mutant, we conclude that expression of the *pilE* G4-associated non-coding sRNA is essential for pilin Av, but that the identity of the polymerase providing the transcription is not important.

### The *pilE* G4-associated sRNA acts in *cis* and its direction and orientation are required for function

Since transcription of the *pilE* G4-associated sRNA allows for melting of duplex DNA containing the *pilE* G4 sequence which then enables formation of the G4 structure, we reasoned that this sRNA might act in-*cis*. Therefore, we determined whether expression of the *pilE* G4-associated sRNA in-*trans* could complement a promoter mutant at the endogenous locus. The G4 sRNA region with a wild-type or stronger promoter was cloned and inserted at the ectopic NICS locus on the chromosome [26] (Fig. S4A). Kinetic pilus-dependent phase-variation assays demonstrated that expression of the sRNA at the ectopic locus does not complement a promoter mutant at the endogenous locus indicating that the *pilE* G4-associated sRNA acts in-*cis* and provides an essential step for the formation of the *pilE* G4 structure (Fig. S4B).

**Figure 4:**
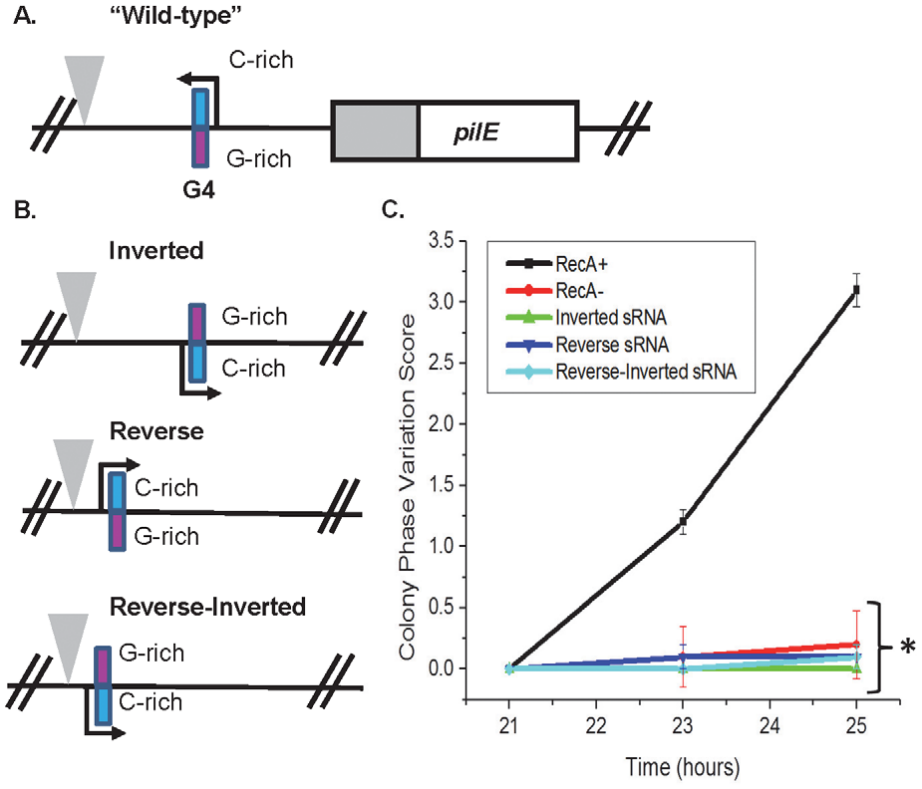
The orientation and direction of the *pilE* G4 sRNA is required for its function. **A.** The *pilE* G4 sRNA region. The C-rich strand (blue) and G-rich strand (pink) are designated. A triangle indicates a transposon insertion used for selection of transformation. **B.** The *pilE* G4 sRNA endogenous locus mutants. A representation of the inverted, reverse, and reverse-inverted sRNA mutants is shown. A triangle indicates a transposon used as a marker for transformation. **C.** Kinetic pilus-dependent colony phase variation assay. The *pilE* G4 sRNA inverted, reverse, and reverse-inverted do not allow pilin Av. *P*<0.05 as determined by two-tailed Student's T-test (asterisks). Error bars represent the standard error of the mean for 10 colonies. A representative assay of *n* = 3 is shown.

Because the *pilE* G4 sRNA acts in-*cis*, we determined whether the orientation and direction of the sRNA at the endogenous locus was also required for its function. Mutants with an inverted *pilE* sRNA, a *pilE* sRNA in reverse, and a *pilE* sRNA that is both in reversed and inverted were created (Fig. 4A&B). None of the mutants allowed pilin Av, indicating that both correct orientation and direction are required for the sRNA and G4 structure to function (Fig. 4C).

## Discussion

**Figure 5:**
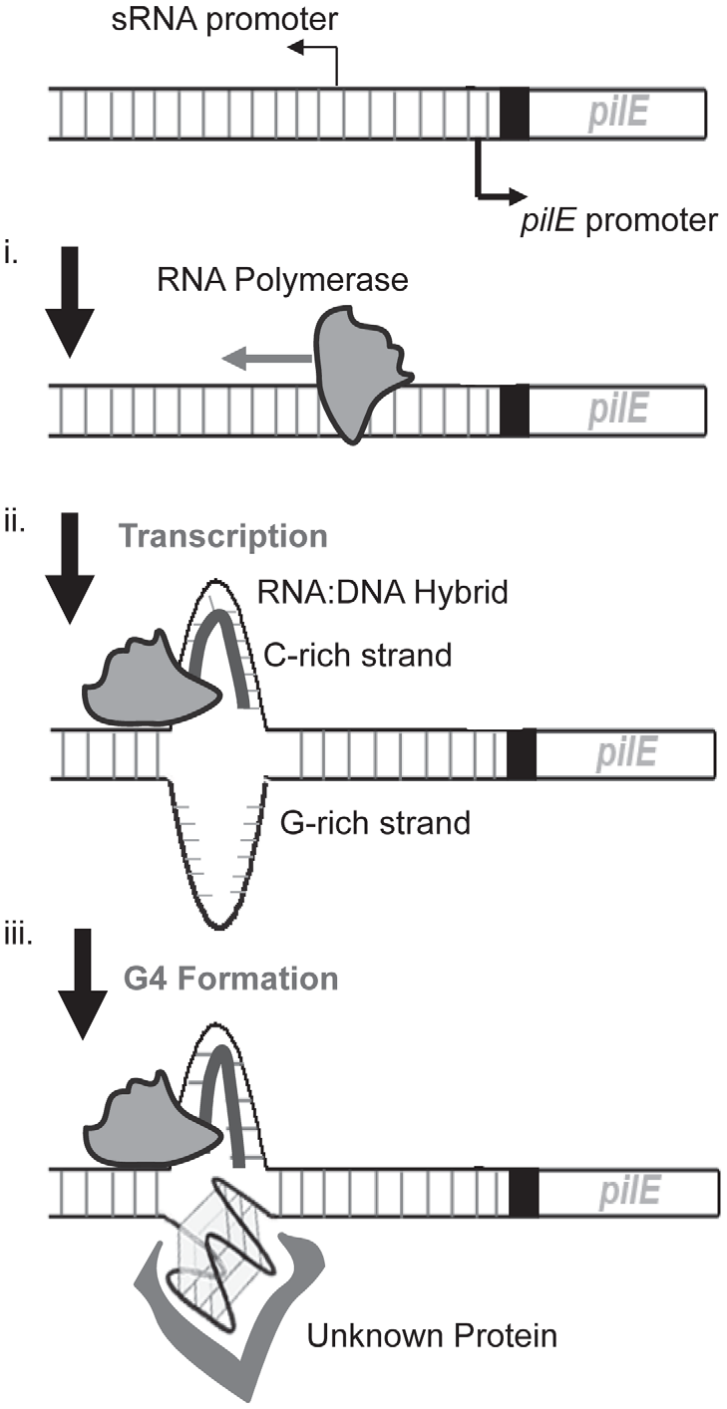
A working model of the initiation of pilin Av in *N. gonorrhoeae*. Shown is the pilin expression locus (*pilE*) and the upstream region containing the *pilE* G4-associated sRNA promoter. We propose that the initiation of pilin Av begins with transcription of the *pilE* G4-associated sRNA promoter (i). During transcription, a RNA∶DNA hybrid is made on the C-rich strand of the *pilE* G4 sequence (ii) facilitating the formation of a G4 structure by the unpaired G-rich strand, which may be mediated by structure specific binding proteins (iii).

We have established a direct link between transcription and G4 structure formation in the process of pilin Av. We show that transcription of the *cis*-acting *pilE* G4-associated non-coding sRNA is absolutely required for *N. gonorrhoeae* pilin Av and is therefore required to initiate the homologous recombination reactions leading to pilin Av. From this work, we can propose a working model for the initiation of pilin Av (Fig. 5). We propose that initiation of pilin Av begins with transcription of the *pilE* G4-associated sRNA. Pilin Av was not enhanced by substitution of the −10 promoter element with a stronger promoter or a phage promoter, which suggests that pilin Av has a maximal level of efficiency that is not influenced by increased *pilE* G4 formation potential. However, since we have not been able to detect the sRNA directly, this conclusion is based on the assumption that these promoters function similarly in *N. gonorrhoeae* as they do in *E. coli*. It was surprising that that transcription of the sRNA by T7 polymerase in *N. gonorrhoeae* resulted in wild type levels of phase variation. This result rules out a direct role for RNA polymerase in G4 structure formation and suggests that it is the act of transcription and/or the sRNA product that are critical for G4 structure formation and pilin Av.

It is also possible that other processes involved in pilin Av downstream of transcription may be rate-limiting, thus negating any effect of increases in transcription rates. We reason that as transcription proceeds through the *pilE* G4 sequence, an RNA∶DNA hybrid could form between the sRNA and the C-rich strand leaving the G-rich strand unpaired to aid G4 structure formation, possibly aided by an as yet unknown protein (Fig. 5). How the formation of a G4 structure then initiates gene conversion is presently unknown, but the affinity for the *pilE* G4 structure by RecA, and the ability of the *pilE* G4 to stimulate RecA-mediated strand exchange [27], supports a model where the G4 structure acts to recruit recombination factors to the *pilE* region of the chromosome. In addition, it is likely that the G4 structure would cause a replication fork collapse when the replisome tries to synthesize past the G4 structure [11].

The lack of any observable pilin Av when the orientation and direction of both the sRNA and G4 structure were changed shows that this element does not act like enhancer elements found in eukaryotic cells. We did not observe any growth defect in strains where the G4-forming sequence was moved from the lagging strand onto the leading strand. This result suggests that either the structure doesn't form often enough to produce an observable reduction in viability when the G4 structure interrupts replication, or there are proteins that can remove the G4 structure to prevent a replication stop. The RecQ helicase is a likely candidate for an enzyme that could remove the G4 structure to prevent a replication blockage (Laty A. Cahoon, Kelly A. Manthei, Ella Rotman, James L. Keck and H. Steven Seifert, unpublished).

Regulatory *cis* and *trans*-encoded small RNAs have been identified in bacteria, but the *pilE* G4-associated sRNA does not fit either the *cis* or *trans*-encoded sRNA paradigm established for bacteria. *Cis*-encoded sRNAs are transcribed antisense to the genes they regulate whereas *trans*-encoded sRNA share limited complementarity. Once base paired with their target mRNA transcript, these regulatory sRNAs can influence the stability of, or modulate translation of, their target [28], [29]. The protein factor Hfq generally binds *trans*-encoded sRNAs and mediates base-pairing of the sRNAs to their target mRNAs; sRNAs that are not bound to Hfq are extremely labile [30]. Generally, *cis*-encoded antisense RNAs (asRNA) do not require Hfq [31]. In *N. gonorrhoeae*, Hfq is an RNA chaperone that functions as a pleiotropic regulator of RNA metabolism [32]. An *hfq* mutant was found to decrease the amount of *pilE* protein by an indirect mechanism that has yet to be determined. Since the *pilE* G4-associated sRNA is not an asRNA or a *trans*-encoded RNA and does not share complementarity to any known mRNA target, it is unlikely that this sRNA binds Hfq, but this has not been experimentally addressed. Some sRNAs have also been shown to bind and regulate proteins, such as the *E. coli* 6S RNA that interacts with RNA polymerase [29].

The *pilE* G4-associated *cis*-acting sRNA is transcribed in a region devoid of open reading frames and is not complementary to any expressed gene. In addition, the sRNA is most likely non-coding since mutations (5–46 bp insertions or 5–32 bp deletions) just downstream of the G-rich region have no effect on pilin Av (Fig. 2 and Fig. S1). It is possible that this sRNA performs other functions in this bacterium and could be processed or form a structure on its own. While we cannot rule out the possibility that the *pilE* G4-associated sRNA regulates other molecules, it is likely that the unwinding of the duplex DNA containing the *pilE* G4 sequence during transcription, and formation of the RNA∶DNA hybrid with the C-rich strand, allows formation of the G4 structure which is required for pilin Av (Fig. S3). It is interesting to note that two other predicted G4 forming sequences in the gonococcal genome also have putative promoters in a similar relative location. Formation of a G4 structure via transcription and occlusion of the C-rich stand has also been previously proposed to act during immunoglobulin class switching [33], [34]. Since both pilin Av and immunoglobulin class switching are high frequency recombination systems, there may exist other diversification systems that use transcriptionally induced alternative DNA structures to initiate programmed recombination reactions, and these may represent a specialized class of transcription-associated recombination systems [35].

While G4 DNA structures have been implicated in many biological processes in eukaryotic cells, less is known about their function in prokaryotic cells. In eukaryotes, G4 structures have been implicated in telomere metabolism [36], the regulation of genes required for normal cell growth and differentiation [37], potentiating genomic instability [38], the regulation of RNA transcription and translation [39], and mediating immunoglobulin gene diversification [33]. Proteins that bind or resolve G4 structures in both eukaryotes and prokaryotes have also been identified [40]–[42]. In prokaryotes, potential G4 forming sequences have been identified by bioinformatics, but functional analysis of these sequences has yet to be reported [43]. Our previous report demonstrating that the *pilE* G4 is essential for pilin Av in *N. gonorrhoeae* was the first study to show the requirement of a G4 structure for any biological process in a bacterial cell [17]. This work develops the mechanism of action further by showing that transcription of a *cis*-acting sRNA is critical for G4 structure formation to direct this specialized diversity generation system.

## Materials and Methods

### Bacterial growth conditions

*E. coli* One Shot TOP10 competent cells (Invitrogen) were grown in Luria-Bertani (LB) broth or on solid media containing 15 g/L agar at 37°C and used to propagate plasmids. *E. coli* selected for plasmids containing kanamycin or erythromycin resistance were selected on media containing 50 or 100 µg/ml of the respective antibiotic. Gonococcal strains were grown on GC Medium Base (Difco) plus Kellogg supplements (GCB) [22.2 mM glucose, 0.68 mM glutamine, 0.45 mM co-carboxylase, 1.23 mM Fe(NO_3_)_3_; all from Sigma] at 37°C in 5% CO_2_, when applicable 1 mM isopropyl β-D-1-thiogalactopyranoside (IPTG) was added for induction. Gonococcal transformants were selected on media containing 50 µg/ml kanamycin or 2 µg/ml erythromycin.

### Pilin Av assays

Gonococci were revived from frozen stocks, then after 24 hours of growth a single piliated colony was passaged onto solid media to obtain single isolated colonies. Colony variation was scored every 2 hours after 18 hours of growth up to 28 hours by observing the number of non-piliatied (P-) sectors arising over time. 10 colonies per strain were scored per assay. A colony that showed no P- sectors was given a score of 0, one P- sector was given a score of 1 and so forth. Colonies with 4 or more P- secors were given a score of 4.

#### 5′ Rapid Amplification of DNA Ends (5′RACE)

Gonococci swabbed from a confluent lawn grown to 20 generations were resuspended (in RNA protect, Qiagen) and harvested for RNA (RNeasy Kit, Qiagen). 1 µg total RNA was treated with DNase I and enriched for non rRNA (using a Microbe Express Kit, Ambion). Then RNA was ethanol precipitated followed by the 5′RACE Protocol adapted for bacterial small non-messenger RNA [23].

### Gonococcal sRNA promoter mutants and kinetic Pilus-dependent colony phase variation

A plasmid containing the region between USS2-pilArev2 from FA1090 (1-81-S2) *pilE*::mTn#9 *recA6* [17] was digested at PacI and Mlu1 sites. Annealed linkers with PacI (TopA/BotA), an overlapping overhang and MluI ends for the −10 promoter mutant (10KOTop/10KOBot), stronger −10 mutant (10strTop/10strBot), T7 promoter mutant (T7G4PTop/T7G4PBot), or PacI and MluI ends for the 5 bp insertion between the −10 and −35 mutant (5in-10-35Top/5in-10-35Bot), were ligated to the PacI/MluI digested plasmid and transformed into TOP10 competent cells (Table S1). The −35 promoter mutant was created by digesting the USS2-pilArev2 plasmid (above) at MluI and EcoRV sites. Then the −35KO linker (35KOTop/35KOBot) was ligated to the digested plasmid. To increase the amount of homologous DNA available for recombination, a plasmid containing the −35 mutation was PCR amplified with GCUUSS2 and a −35 lengthening primer using KOD (Novagen) and cloned into pCRBluntII-Topo (Table S1). To create the −10 and −35 promoter mutant, a plasmid containing the −35 promoter mutation was digested with PacI and MluI. Then, the linker used to create the −10 promoter mutant was ligated to the digested plasmid (Table S1). Then gonococcal strain FA1090 (variant 1-81-S2) *recA6* was transformed as described previously [44] and selected for kanamycin resistance. Kinetic phase variation assay was performed as described [16].

### Gonococcal promoter region mutants

The USS2-PilArev2 plasmid was digested with PacI and MluI. Annealed linkers with MluI (G4Bot/G4Top), an overlapping overhang and PacI ends for the 10 bp insertion or deletion downstream of the G4 (in10bpDTop/in10bpDBot and del10bpDTop/del10bpDBot, respectively), the 5 bp insertion or deletion downstream of the G4 (in5bpDTop/in5bpDBot and del5bpDTop/del5bpDBot, respectively), were ligated to the PacI/MluI digested plasmid (Table S1). The 5 bp insertion upstream of the sRNA promoter mutant was created by digestion of the USS2-pilArev2 plasmid at unique MluI and EcoRV sites follow by ligation to the linker (in5bpUTop/in5bpUBot) (Table S1). Then gonococcal strain FA1090 (1-81-S2) *recA6* was transformed as described [44] and selected for kanamycin resistance. Other mutants (Fig. 2, the 9 bp deletion and 46 bp insertion downstream of the G4) were identified during screens for the desired mutations listed above. Point mutants (Fig. 2) were identified in a previous screen [17] and backcrossed into the parental strain and selected for kanamycin resistance.

### *In vitro* transcription and primer extension analysis

For *E. coli* RNA polymerase (holoenzyme, sigma saturated, Epicentre) *in vitro* transcription reactions, template DNA was PCR generated using KOD, gonococcal DNA having the desired *pilE* G4 sRNA region mutation, and primers RS1For and pilARevnested2Bot (Table S1). Then using 100 ng of purified template, *in vitro* transcription reactions were performed as specified by the manufacture. After 1–2 hours at 37°C, reactions were DNase I treated and run on a 14% polyacrylamide denaturing gel (UreaGel System, National Diagnostics). 0.5 µl of the above reactions were used for primer extension analysis, cDNA was synthesized using 3 pmol of 5′FAM-labeled PEx1 primer and Superscript III, EtOH precipitated, and sent for fragment analysis (Northwestern Genomics Core Facility). For T7 RNA polymerase *in vitro* transcription reactions, template DNA was PCR generated using KOD, gonococcal DNA having the replacement T7 promoter at the *pilE* G4-associated sRNA locus, and primers RS1For and T7G4invitro (Table S1). Then using 100 ng of purified template, *in vitro* transcription reactions were performed (Maxiscript Kit, Ambion).

### The T7 promoter/T7 polymerase strain

Creation of the *pilE* G4-associated sRNA T7 promoter replacement mutant is detailed above. To create the IPTG inducible T7 polymerase strain, we used KOD and primers PacIT7F engineered to have a Pac I site and T7R to amplify T7 polymerase from BL21 (DE3) *E. coli* (Table S1). This PCR product was digested with PacI and cloned into the PacI and PmeI digested pGCC4 vector [26]. This construct was allowed to recombine into strain FA1090 (1-81-S2) at the NICS by liquid transformation and was selected for on media containing erythromycin [26], [45], [46]. Then chromosomal DNA from this strain was transformed into FA1090 (1-81-S2) *recA6*, and the *pilE* G4-associated sRNA T7 promoter replacement mutant. These mutants were then assayed for pilus-dependent colony phase variation [16].

### Transformation of the Holliday junction processing mutant

The −10 and −35 *pilE* G4-associated sRNA promoter mutant plasmid detailed above was allowed to recombine into the Holliday junction processing mutant by liquid transformation [25], [46]. After limited exposure to IPTG, colonies were selected for kanamycin resistance, tested for erythromycin resistance associated with the *ruvB* functional deletion and chloramphenicol associated with the *recG* functional deletion. Gonococcal transformants containing the −10 and −35 sRNA promoter mutations were verified by sequencing analysis.

### The *pilE* G4 sRNA associated ectopic expression strain

The region from the edge of the transposon insertion in RS1 which does not affect pilin Av [15] to 25 bps upstream of the *pilE* G4-associated sRNA −35 promoter element were PCR amplified using KOD and primers G4compR1PacI engineered to have a Pac I site and RS1For (Table S1). This PCR product was digested with PacI and cloned into the digested PacI and PmeI pGCC4 vector. This construct was allowed to recombine into the *pilE* G4-assoicated sRNA −10 promoter mutant strain at the NICS as described previously [44] and selected for erythromycin resistance. Transformants were verified by sequencing both the ectopic and endogenous *pilE* G4-assoiciated sRNA site. These mutants were then assayed for pilus-dependent colony phase variation [16].

### The *pilE* G4-associates sRNA direction and orientation mutants

The sRNA inverted, reverse, reverse-inverted and respective −10 and −35 promoter element mutants were created by ligating a PacI digested 433 bp synthesized dsDNA fragment (IDT) to the PacI/EcoRV digested USS2-pilArev2 plasmid (Table S1). Then gonococcal strain FA1090 (1-81-S2) *recA6* was transformed as described [44] and selected for kanamycin resistance.

## Supporting Information

Figure S1 **Kinetic pilus-dependent colony phase variation assays for mutants shown in** **Figure 2** **.** **A.** Promoter −10 and −35 point mutants. **B.** Mutants with 5 bp insertions upstream of the −35 or between (btwn) the −10 and −35 promoter elements and mutants with point mutations between the −10 and −35 promoter elements. **C.** Mutants with a 10 bp insertion or deletion downstream of the *pilE* G4. **D.** Mutant with a 9 bp deletion downstream of the *pilE* G4. **E.** Mutant with a 46 bp insertion downstream of the *pilE* G4. **F.** Mutants with a 5 bp insertion or deletion downstream of the *pilE* G4. *P*<0.05 as determined by two-tailed Student's T-test (asterisks). Error bars represent the standard error of the mean for 10 colonies. A representative assay of *n* = 3 is shown.
(EPS)

Figure S2 **Primer extension analysis.** cDNA was synthesized from in vitro transcribed RNA using a FAM-labeled primer initiating in RS1. Plots from the wild-type promoter, stronger promoter and mutant −10 and −35 are shown. Relative fluorescence (RF) is plotted over length (L) where the primer and primer extension product peak are indicated.
(EPS)

Figure S3 **Mutation of the** ***pilE*** **G4-associated sRNA promoter rescues the synthetic lethality of the Holliday processing mutations.** Colonies were grown for 25 hours with or without IPTG to control for RecA induction. **A.** RecA- colonies: The parental, *recG/ruvB*, and *recG/ruvB/*mt-10-35 strains grown in the absence of RecA induction are similar in growth. **B.** RecA+ colonies: The *recG/ruvB* Holliday junction processing mutant previously characterized shows a synthetically lethal phenotype upon RecA induction S [31]. Mutation of the *pilE* G4-associated sRNA promoter (mt-10-35) rescues the synthetically lethal phenotype of the *recG/ruvB* double mutant which replicates the phenotype previously shown for a *pilE* G4 mutant [1].
(EPS)

Figure S4 **Expression of the** ***pilE*** **G4-associated sRNA at an ectopic locus does not rescue the sRNA promoter mutant phenotype.** **A.** The pilE G4-associated sRNA complement strain. The G4 sRNA region (upstream of the −35 element and 75 bp downstream of +1) was cloned downstream of a dual *taclac* promoter which allows induction with the addition of IPTG [32]. The construct was transformed into a *pilE* G4-associated sRNA −10 promoter mutant and inserted into the NICS [26]. [A triangle indicates a kanamycin transposon (TN) that was used as a marker for transformation to create the sRNA promoter mutant]. **B.** Kinetic pilus-dependent colony phase variation assay. The p*ilE* G4-associated sRNA containing a wild-type (G4 sRNA wt-10) or stronger (G4 sRNA str-10) promoter expressed at an ectopic site (NICS) does not rescue the phenotype of the sRNA −10 mutant (mt-10). *P*<0.05 as determined by two-tailed Student's T-test (asterisks). Error bars represent the standard error of the mean for 10 colonies. A representative assay of *n* = 4 is shown.
(EPS)

Table S1 **Oligonucleotides used in this study.**
(DOCX)
