# Supplementary material for: Transcription of a cis-acting, Noncoding, Small RNA Is Required for Pilin Antigenic Variation in Neisseria gonorrhoeae
Source: PLoS Pathog. 2013 Jan 17;9(1):e1003074. doi: 10.1371/journal.ppat.1003074 (PMC3547852; doi:10.1371/journal.ppat.1003074)
Supplement: Table S1 — Oligonucleotides used in this study. (DOCX) [file ppat.1003074.s005.docx]

Table S1

| Oligo | Sequence (5’-3’) | Use |
| --- | --- | --- |
| RNA Adapter | AUAUGCGCGAAUUCCUGTAGAACGAACACUAGAAGAAA | RACE |
| RS1F2 | GCTTTCCAAGAAAACGGAGCTT | RACE |
| Adapter Specific | GCGCGAATTCCTGTAGA | RACE |
| RS1F3 | CCAAGAAAACGGAGCTTTTTAA | RACE |
| TopA | TAAACCACGCGCCGATTTCAAATGCTTTCCAAGAAAACGGAGCTTT | Promoter mutants linker |
| BotA | P^1^_TCCGTTTTCTTGGAAAGCATTTGAAATCGGCGCGTGGTTTAAT | Promoter mutants linker |
| 10KOTop | P_TTAAAAAATAAAAAATTCCCCACCCAACCCACCCTGGACGGA | Promoter -10KO linker |
| 10KOBot | CGCGTCCGTCCAGGGTGGGTTGGGTGGGGAATTTTTTATTTTTTAAAAAGC | Promoter -10KO linker |
| 10strTop | P_TTAAAAAATAAAAAATTCCCCACCCAACCCACCCTATTATAA | Stronger -10 Linker |
| 10strBot | CGCGTTATAATAGGGTGGGTTGGGTGGGGAATTTTTTATTTTTTAAAAAGC | Stronger -10 Linker |
| T7G4PTop | P_TTAAAAAATAAAAAATTCCCCACCCAACCCACCCTATAGTGAGTCGTATTA | T7 minimal promoter linker |
| T7G4PBot | CGCGTAATACGACTCACTATAGGGTGGGTTGGGTGGGGAATTTTTTATTTTTTAAAAAGC | T7 minimal promoter linker |
| 5in-10-35Top | TAAACCACGCGCCGATTTCAAATGCTTTCCAAGAAAACGGAGCTTTTTAAAAAATAAAAAATTCCCCACCCAACCCACCCTATTCTATAAGGA | 5 bp insertion between -10 and -35 promoter elements linker |
| 5in-10-35Bot | CGCGTCCTTATAGAATAGGGTGGGTTGGGTGGGGAATTTTTTATTTTTTAAAAAGCTCCGTTTTCTTGGAAAGCATTTGAAATCGGCGCGTGGTTTAAT | 5 bp insertion between -10 and -35 promoter elements linker |
| 35KOTop | CGCGTAAATTCAAAAAGAGTGGATTCCGACCCAATCAACAC | Promoter -35KO linker |
| 35KOBot | GTGTTGATTGGGTCGGAATCCACTCTTTTTGAATTTA | Promoter -35KO linker |
| 35 lengthening | ACTTTTTTATTGGCATGGGGTATCGGGTGTGTTGATTGGG | Promoter -35KO lengthening primer |
| Kan4 | CAAGGGGTGTTATGAGCCAT | PCR and sequencing the region between RS1 and pilA |
| GcuKpnPilArev2 | GCACTAAAACTGACAATTTTCGACACTGCCGCCTTCAGACGGCGGTACT | PCR and sequencing the region between RS1 and pilA |
| GCUUSS2 | GCCGTCTGAACCAACTGCCACCTAAGG | Cloning PCR |
| PilARevNested2Bot | GTCAGTTTTAGTGCCGATTTTCG | Template PCR for *in vitro* transcription |
| PEx1 | FAM^2^- CCACGCGCCGATTTCAAATG | cDNA synthesis for fragment analysis |
| T7G4invitro | TAATACGACTCACTATAGGGTGGGTTGGGTGGGG | Template PCR for *in vitro* transcription |
| PacIT7F | ATACTTAATTAAATGAACACGATTAACATCGC | Amplification and cloning of T7 RNA polymerase |
| T7R | TTACGCGAACGCGAAGTCCGA | Amplification and cloning of T7 RNA polymerase |
| T7F2 | GACATGCTCTCTAAGGGTCT | PCR and sequencing T7 RNA polymerase |
| T7F3 | GAGGACATCCCTGCGATTGA | PCR and sequencing T7 RNA polymerase |
| T7F4 | CTAGTGAGACCGTTCAGGAC | PCR and sequencing T7 RNA polymerase |
| T7F5 | CCTGATGTTCCTCGGTCAGT | PCR and sequencing T7 RNA polymerase |
| G4compR1PacI | ATACTTAATTAATCGGGTGTGTTGATTGGGTC | Creating the G4 ectopic locus strain |
| RS1For | ACACCACGCGCCGATTTCAAATG | Creating the G4 ectopic locus strain |
| G4Top | P_TTAAAAAATAAAAAATTCCCCACCCAACCCACCCTATTCTAA | Promoter mutants linker |
| G4Bot | CGCGTTAGAATAGGGTGGGTTGGGTGGGGAATTTTTTATTTTTTAAAAAGC | Promoter mutants linker |
| in10bpDTop | AAACCACGCGCCGATTTCAAATGCTTTCCAAGAAAACAACGGTAAGGGGAGCTTT | 10 bp insertion downstream of G4 linker |
| in10bpDBot | P_TCCCCTTACCGTTGTTTTCTTGGAAAGCATTTGAAATCGGCGCGTGGTTTAAT | 10 bp insertion downstream of G4 linker |
| in5bpDTop | TAAACCACGCGCCGATTTCAAATGCTTTCCAAGAAAACTAAGGGGAGCTTT | 5 bp insertion downstream of G4 linker |
| in5bpDBot | P_TCCCCTTAGTTTTCTTGGAAAGCATTTGAAATCGGCGCGTGGTTTAAT | 5 bp insertion downstream of G4 linker |
| del10bpDTop | TAAACCACGCGCCGATTTCAAATGCTTTGGAGCTTT | 10 bp deletion downstream of G4 linker |
| del10bpDBot | P_TCCAAAGCATTTGAAATCGGCGCGTGGTTTAAT | 10 bp deletion downstream of G4 linker |
| del5bpDTop | TAAACCACGCGCCGATTTCAAATGCTTTAAAACGGAGCTTT | 5 bp deletion downstream of G4 linker |
| del5bpDBot | P_TCCGTTTTAAAGCATTTGAAATCGGCGCGTGGTTTAAT | 5 bp deletion downstream of G4 linker |
| in5bpUTop | CGCGTAAATTCAAAAATCTCAAATTCCGACCTAAGGCAATCAACACACCCGATACCCCATGCCATTCAGACGGC | 5 bp insertion upstream of G4 linker |
| in5bpUBot | GCCGTCTGAAtGGCATGGGGTATCGGGTGTGTTGATTGCCTTAGGTCGGAATTTGAGATTTTTGAATTTA | 5 bp insetion upstream of G4 linker |
| G4RNAGinv | ATACTTAATTAACGTATCTTTGTGGTGCGCGGCTAAAGTTTACGAAAGGTTCTTTTGCCTCGAAAAATTTTTTATTTTTTAAGGGGTGGGTTGGGTGGGATAAGATTGCGCATTTAAGTTTTTAGAGTTTAAGGCTGGGTTAGTTGTGACCCGATACCCCATGCCAATAAAAAAGTAACGAAAATCGGCACTAAAACTGACAATTTTCGACACTGCCGCCCCCTACTTCCGCAAACCACACCCACCTAAAAGAAAATACAAAATAAAAACAATTATATAGAGATAAACGCATAAAATTTCACCTCAAAACATAAAATCGGCACGAATCTTGCTTTATAATACGCAGTTGTCGCAACAAAAAACCGATGGTTAAATACATTGCATGATGCCGATGGCGTAAGCCTGAGGCATTTCCCCTTTCGCCGTCTGAACC | PacI site 3’ gonococcal uptake sequence containing dsDNA fragment used to create the inverted *pilE* G4-associated sRNA construct |
| G4RNACrev | ATACTTAATTAACACAACTAACCCAGCCTTAAACTCTAAAAACTTAAATGCGCAATCTTATCCCACCCAACCCACCCCTTAAAAAATAAAAAATTTTTCGAGGCAAAAGAACCTTTCGTAAACTTTAGCCGCGCACCACAAAGATACGACCCGATACCCCATGCCAATAAAAAAGTAACGAAAATCGGCACTAAAACTGACAATTTTCGACACTGCCGCCCCCTACTTCCGCAAACCACACCCACCTAAAAGAAAATACAAAATAAAAACAATTATATAGAGATAAACGCATAAAATTTCACCTCAAAACATAAAATCGGCACGAATCTTGCTTTATAATACGCAGTTGTCGCAACAAAAAACCGATGGTTAAATACATTGCATGATGCCGATGGCGTAAGCCTGAGGCATTTCCCCTTTCGCCGTCTGAACC | PacI site 3’ gonococcal uptake sequence containing dsDNA fragment used to create the reverse *pilE* G4-associated sRNA construct |
| G4RNAGrevinv | ATTGTTAATTAAGTGTTGATTGGGTCGGAATTTGAGATTTTTGAATTTACGCGTTAGAATAGGGTGGGTTGGGTGGGGAATTTTTTATTTTTTAAAAAGCTCCGTTTTCTTGGAAAGCATTTGAAATCGGCGCGTGGTGTTTCTATGCACCCGATACCCCATGCCAATAAAAAAGTAACGAAAATCGGCACTAAAACTGACAATTTTCGACACTGCCGCCCCCTACTTCCGCAAACCACACCCACCTAAAAGAAAATACAAAATAAAAACAATTATATAGAGATAAACGCATAAAATTTCACCTCAAAACATAAAATCGGCACGAATCTTGCTTTATAATACGCAGTTGTCGCAACAAAAAACCGATGGTTAAATACATTGCATGATGCCGATGGCGTAAGCCTGAGGCATTTCCCCTTTCGCCGTCTGAACC | PacI site 3’ gonococcal uptake sequence containing dsDNA fragment used to create the reverse-inverted *pilE* G4-associated sRNA construct |

^1^ P_ indicates a phosphorylated linker used for cloning.
^2^ FAM represents a 6-carboxyfluorescein label.
